# Nutritional policies and anaemia among under-five children in selected south Asian countries: 1950–2016

**DOI:** 10.1186/s12887-022-03597-4

**Published:** 2022-09-12

**Authors:** Hanumant Waghmare, Nasim Ahamed Mondal, Babul Hossain

**Affiliations:** 1 Department of Migration and Urban Studies, Post-Doctoral Fellow, International Institute for Population Sciences, Mumbai, Maharashtra Pin: 400088 India; 2 Department of Social Sciences, Research Scholar, University of Warsaw, Warsaw, Poland; 3 Centre of Migration Research, Research Associate, University of Warsaw, Warsaw, Poland; 4 Doctoral Fellow, International Institute for Population Sciences, Mumbai, Maharashtra Pin: 400088 India

**Keywords:** Nutritional Policies, Anaemia, Nutritional Status, Under-5 children, South Asia

## Abstract

**Background:**

Despite policies and social development, childhood malnutrition remains a significant public health and social challenge in many south Asian countries. Also, there is a lack of study for a comparative understanding between the nutrition policies and nutritional status of under-5 children in south Asian countries. In this context, the present study aims to understand the comparative evolution of nutritional policies and programs in south Asian countries between 1950 to 2016 and assess current nutritional status of children under the age of 5 in Bangladesh, India, Nepal, and Pakistan.

**Methods:**

This study obtained yearly anaemia data from the Global Health Observatory Data Repository (World Health Statistics) from 1990 to 2016 for comparative analysis. The most recent Demographic Health Survey (DHS) rounds have been taken for four south Asian nations. Bivariate analysis and classical figures have been utilised to demonstrate the findings.

**Results:**

In Bangladesh, Nepal, India, and Pakistan, the prevalence of anaemia decreased by 33%, 31%, 20%, and 12% from 1990 to 2016, respectively. While analysis of the policy and programs, the problem of under-nutrition in all selected countries stems from the lack of serious implementation of National Nutrition Policies.

**Conclusion:**

This study suggests that the national nutrition programs require inter-sectoral coordination between central ministries within countries to reduce the anaemia level. In light of Sustainable Development Goals (SDG), a multi-faceted policy should be implemented to prevent and control malnutrition problems in these countries.

## Background

World Health Organisation (2020) defines malnutrition includes undernutrition, inadequate vitamins or minerals, overweight, obesity, and resulting diet-related non-communicable illnesses [1]. The consequences of inadequate nutrition begin in the womb, persist long into adulthood, and are passed down through generations [2]. Malnutrition is a major cause of morbidity and mortality in under-5 children [3, 4]. Around 45% of mortality among children below five years of age is associated with undernutrition, and these mainly occur in low- and middle-income nations [1]. Despite multiple nutritional policies, anaemia affects an estimated 165 million malnourished children under five years in low- and middle-income countries [5]. It is also estimated that globally 30% of children under five are moderately or severely stunted, 19% are moderately or severely underweight, and almost 50% of all stunned children reside in Asia, 8% of under-5 children are wasted worldwide, and two-thirds of all wasted children live in Asia [6].

Malnutrition results from low food intake and is related to transmission factors such as crowding, water, and environmental hygiene [7]. Childhood malnutrition is attributed to low birth weight, insufficient breastfeeding and exclusive breastfeeding, inappropriate complementary feeding, maternal education, lack of proper micronutrient intake, parity, birth spacing, socioeconomic level of the home, food insecurity, inadequate sanitation, immunization, and infectious illnesses [8–10].

The leaders from 159 countries in attendance at 1992 International Conference on Nutrition in Rome declared their determination to eradicate hunger and decrease all forms of malnutrition. Soon after this, Industrialized and developing countries have started to formulate and implement food and nutrition-related policies [11]. With economic development, nutrition policies have evolved to eradicate malnutrition among children [4, 12], but malnutrition rates have decreased slightly, and progress is plodding. The situation is worst in India, Pakistan, Bangladesh, and Nepal, which failed to reduce the required level of malnourishment, even after a long history of nutritional policy commitment to combat anaemia among children. Despite policies and social development, childhood malnutrition remains a significant public health and social challenge in less developed countries [10].

To the factors of malnutrition, South Asian countries adopted different policies and programs over the periods. For every country, nutrition status constitutes the foundation for human development by reducing susceptibility to infections, disability, morbidity, and mortality burden, enhancing cumulative lifelong learning capacities, and adult efficiency [13]. Nutrition is widely recognised as a very effective entry point for human development, poverty alleviation, and economic growth, with significant economic rewards [14].

A large body of literature in four selected South Asian countries have examined the trend, pattern, prevalence, and factors for child anaemia and nutrition in a broader context [15–22]. However, there is a lack of study for a comparative understanding between the nutrition policies and nutritional status of under-5 aged children in South Asian countries. This study provides an overview of nutrition policies and actions taken to improve the nutritional status of under-five children in the South Asian context, focusing on Bangladesh, India, Nepal, and Pakistan.

The countries have a unique context; each is in different development stages in terms of nutrition policies and programs and has adopted varying approaches to strengthening their children's nutrition services. The countries provide a rich insight into what's being done to improve children's nutrition status and what more needs to be done in the future. Yet, for South Asia, children's nutrition is very much unfinished business, and, as the countries themselves acknowledge, more improvement is required to eradicate anaemia. In this context, the present study aims to understand the comparative evolution of nutritional policies and programs in South Asian countries and their impact on children's nutritional status.

## Methods

### Data source

The study collected year-wise anaemia data from 1990 to 2016 from World Health Organization, Global Health Observatory Data Repository (World Health Statistics) for comparative analysis [23]. The latest round of Demographic Health Survey (DHS) data from four South Asian countries: India 2015–16 (NFHS-4) (*N* = 259,627), Nepal 2016 (NDHS-5) (*N* = 5,038), Pakistan 2012–13 (PDHS-3) (*N* = 12,708), and Bangladesh 2014 (BDHS-7) (*N* = 7,886) used to study the distribution of children according to background characteristics in selected South Asian countries. DHS is a nationally representative sample covering samples from across the countries with a well-specified sampling procedure. All the DHS uses multi-stage stratified sampling for sample selection. India had the largest sample size out of the four selected countries, and Nepal had the smallest sample size.

### Data analysis and policy document review

Bivariate analysis was used to study the distribution of children according to background characteristics in selected South Asian countries. To review the nutritional policies in selected South Asian countries, we collected literature from various websites, journals, and documents. We investigated constitutional provisions by multiple countries for nutritional support through the literature. The selected countries have made efforts to improve their nutritional status by implementing various programs and policies. Thus, we identified essential policies and studied their nature, guideline, and resolutions, and chronological implantation by India, Pakistan, Nepal, and Bangladesh. The specified policies and programs are plotted on graphs with the prevalence of anaemia.

We also pooled (combination of selected countries data) the data of four South Asian countries to give an inkling of the overall distribution of children according to background characteristics in South Asia. This study considers anaemia for showing its historical changes over time since we could gather year-wise historical data on anaemia only.

The selected socio-economic and demographic factors for the distribution of under-five children in selected South Asian countries are: Child age in the months (0–12, 13–24, 25–36, 37–48 and 49–60), Place of residence (Rural, Urban), Educational attainment of the mother (No education, Primary, Secondary and Higher), Sex of the Household head (Male and Female), Source of drinking water (Piped water, Tubewell/borewell, Protected well, Unprotected well, River/dam/springs and Others), Type of fuel used for cooking (Clean, Wood, Crop residual, Animal dung and Others), Type of toilet Facilities (Flush toilet, Pit latrine, Open and Other), Wealth quintile (Poorest, Poorer, Middle, Richer and Richest).

## Result

### Demographic profile of the respondent

Table 1 demonstrates the percentage distribution of children according to background characteristics in selected South Asian countries. There is an even distribution of children in each age group (approximately 20%) except 49–60 months (19%) in pooled data. In Bangladesh, the below 12-month age group has the highest percentage of children (21%), whereas the 49–60 months age group has the lowest percentage (18%) of children. In India, each age group has approximately the same percentage of children (about 20%) except the 49–60 months age group (21%). Nepal has a minimum percentage of children in the 25–36 month age group, while Pakistan has the highest percentage of children in the below 0–12 month age group (21%).

**Table 1:** Percentage distribution of children according to background characteristics in selected South Asian countries

Characteristics	Pooled		Bangladesh	India	Nepal	Pakistan
**Child age in month**						
00–12	20.97		21.21	20.93	20.16	21.09
13–24	20.02		20.95	19.92	20.50	19.79
25–36	20.00		20.37	19.99	18.94	19.90
37–48	20.52		19.79	20.86	20.19	19.77
49–60	18.49		17.70	18.30	20.21	19.44
**Place of residence**						
Urban	28.97		25.41	28.05	53.96	31.93
Rural	71.03		74.59	71.95	46.04	68.07
**Educational attainment mother**						
No education	32.38		16.41	30.06	34.25	49.35
Primary	16.21		27.98	14.05	20.14	16.63
Secondary	40.60		46.29	45.41	31.95	21.45
Higher	10.81		9.32	10.48	13.66	12.57
**Sex of household H**						
Male	88.08		91.33	87.74	71.19	88.95
Female	11.92		8.67	12.26	28.81	11.05
**Source of drinking water**						
Piped water		31.12	8.80	35.76	42.34	26.68
Tubewell/borewell		51.02	79.60	45.16	41.84	55.91
Protected well		2.11	0.18	2.55	0.62	1.82
Unprotected well		3.32	0.45	4.44	1.03	1.30
River/dam/springs		2.12	1.55	1.44	4.16	4.64
Others		10.31	9.42	10.65	10.02	9.65
**Type of fuel used for cooking**						
Clean	31.99		14.67	32.10	23.11	42.25
Wood	43.79		47.35	42.50	59.56	44.80
Crop residual	7.82		21.81	6.97	2.84	3.27
Animal dung	8.27		6.65	9.77	6.70	4.15
Others	8.13		9.52	8.65	7.79	5.53
**Type of toilet facilities**						
Flush toilet	46.43		16.13	42.33	62.56	76.36
Pit latrine	13.24		68.53	6.53	8.49	5.53
Open	33.12		2.74	44.10	19.95	13.51
Other	7.21		12.59	7.04	9.23	4.60
**Wealth quintile**						
Poorest	24.48		22.71	25.36	21.39	22.70
Poorer	21.27		19.15	21.98	21.19	20.05
Middle	20.10		19.36	19.83	22.16	20.75
Richer	18.54		19.81	18.12	20.48	19.06
Richest	15.71		18.98	14.70	14.78	17.44
**Total (N)**	100(285,259)		100(7,886)	100(259,627)	100(5,038)	100(12,708)

**Source:** Demographic and health survey: Bangladesh- 2014, India-2015–16, Pakistan-2017–18, Nepal-2016

If we look at the percentage distribution of children by residence, most live in rural areas (71%). Bangladesh, India, and Pakistan show the same trend except for Nepal regarding country-wise distribution. In Nepal, 54% of children live in an urban area. In the mother's education, 32% are illiterate, whereas 16% reported primary education, 40% of mothers said they have secondary education, and 11% reported higher qualifications. Approximately 46% of mothers from Bangladesh and India had secondary qualifications. Remarkably, almost 50% of mothers in Pakistan have reported no education.

Most of the families have a male head (88%). More than 90% reported male head of the household in Bangladesh, followed by India (88%), Pakistan (89%), and Nepal (71%), respectively. More than 50% of families have a tube well/bore well as a drinking water source. Thirty-one percent of families are using piped water as drinking water. In Bangladesh, 80% of families use tube well/ bore well as a drinking water source. In India, Tube well/ bore well (45%) and Piped water (36%) are the primary drinking water sources. Four to five percent of Nepal and Pakistan families use River/ Dam/ Springwater as drinking water. At the same time, 4% of India's families consume drinking water from unprotected wells.

Wood is the first choice as a cooking fuel in all counties. Overall, 44% of families use wood fuel for cooking. Crop residual is used as cooking fuel in Bangladesh (22%). About 60% of families from Nepal use wood as cooking fuel. About 46% of families use flush toilet facilities when it comes to toilet facilities, whereas 33% use Open defecation. Most families use flush toilet facilities except Bangladesh families (only 16%). About 76% of families in Pakistan use flush toilet facilities, which is the highest among all countries. Around 68% of families are using pit toilet facilities in Bangladesh. The highest percentage of families reported open defecation in India (44%). Approximately 24% of families belong to the poorest wealth quintile, followed by the poorer (21%), Middle (20%), Richer (19%), and richest (16%), respectively, in pooled data of selected countries.

### Evolution of nutritional policies and anaemia prevalence in selected South Asian countries

Nutrition is currently relevant in South Asia's political agenda, and most countries are implementing multi-sector national nutrition policies and plans to meet global nutrition targets. Here we describe the probable effect of historical nutrition policies and programs in four selected South Asian countries (Bangladesh, India, Nepal, and Pakistan) individually in light of children’s anaemia in the subsequent paragraphs.

**Fig. 1:**
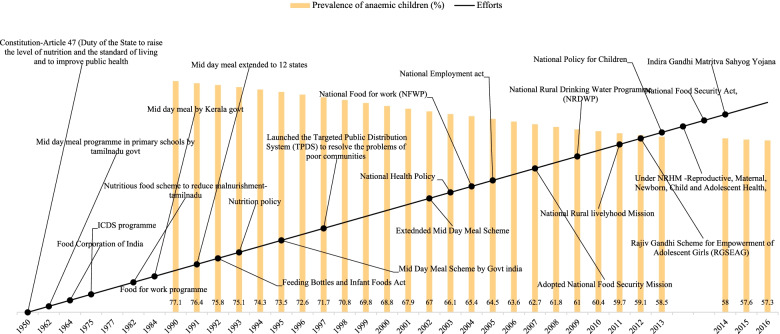
Prevalence of anemia among children aged 0–59 months and the chronology of various efforts to improve their nutritional status, India: 1950–2016

Figure 1 shows the prevalence of anaemia among children aged 0–59 months and the chronology of various efforts to improve India's nutritional status. Figure 1 shows a 77% prevalence of anaemia among children aged 0–59 months in 1990. It also shows that 69%, 60%, and 57% prevalence of anaemia among children in 2000, 2010, and 2016, respectively. Only a 20% points reduction took place in 26 years between 1990 and 2016 in India.

In India, despite a strong constitutional Article 47-Duty of the State to enhance nutrition, living conditions, and public health, significant levels of maternal and child undernutrition have persisted. The government began programmes such as Integrated Child Development Services (ICDS) in 1975, the mid-day meal in 1984, and the nutrition policy in 1993 under this provision. The 2013 National Food Security Act establishes mandatory food and nutrition entitlements for children, pregnant and nursing mothers, and women in need of maternity care. The Substitutes for Infant Milk, Feeding Bottles, and Infant Foods Act 1992 and Amendment Act 2003 provide a strong policy framework for safeguarding, supporting, and promoting nutrition interventions, particularly during children's and women's times of greatest vulnerability.

**Fig. 2:**
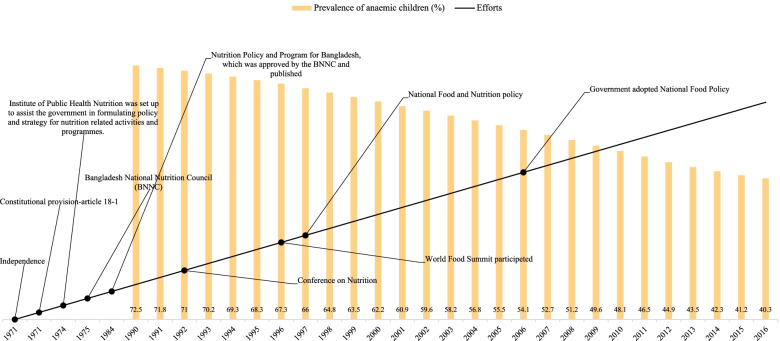
Prevalence of anemia among children aged 0–59 months and the chronology of various efforts to improve their nutritional status, Bangladesh: 1971–2016

Figure 2 shows the prevalence of anaemia among children aged 0–59 months and the chronology of various efforts to improve Bangladesh's nutritional status. Figure 2 displays a 73% prevalence of anaemia among children in 1990. It also shows that 62%, 48%, and 40% prevalence of anaemia among children in 2000, 2010, and 2016 respectively. There is a 33% points reduction in 26 years between 1990 and 2016 in Bangladesh.

Bangladesh's 1972 constitution was the first step toward a more targeted nutrition strategy. The high rates of maternal undernutrition in Bangladesh have continued despite a critical constitutional clause in the 1972 constitution that designated the State's main obligation to enhance individuals' nutritional health. According to Article 18–1 of the Bangladesh constitution, "the State should view increasing the level of nutrition and improving public health as among its major responsibilities" (Shahan & Jahan, 2017). As a result, attempts were undertaken to develop policies that would assist the State in carrying out its obligations. The Institute of Public Health Nutrition was established in 1974 to assist the government in developing nutrition-related policies and strategies. This was followed in 1975 by the Bangladesh National Nutrition Council (BNNC) formulation (Mannan, 2003), a National Food and Nutrition Policy in 1997, and a National Food Policy in 2006 (Shahan & Jahan, 2017).

**Fig. 3:**
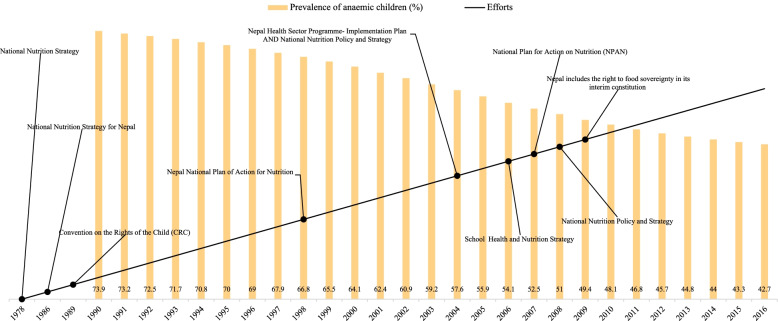
Prevalence of anemia among children aged 0–59 months and the chronology of various efforts to improve their nutritional status Nepal: 1978–2016

Figure 3 shows the prevalence of anaemia among children aged 0–59 months and the chronology of various efforts to improve Nepal's nutritional status. Figure 3 manifests a 74% prevalence of anaemia among children in 1990. It also shows that 64%, 48%, and 43% prevalence of anaemia among children in 2000, 2010, and 2016. There was a 31% points reduction in 26 years between 1990 and 2016 in Nepal.

Nepal undertook a variety of measures to address the country's nutritional concerns. It is a basic human right entrenched in the 1989 United Nations Convention on the Rights of the Child (CRC). Following the 1978 National Nutrition Strategy. Additionally, the Nepal National Nutrition Plan of Action 1998, the Nepal Health Sector Programme-Implementation Plan 2004–2009, the National Nutrition Policy and Strategy 2004, the National Plan for Action on Nutrition (NPAN) 2007, the National Nutrition Policy and Strategy 2008, and the current National Nutrition Program (Pahari, 2011) have been implemented. Furthermore, the government launched the School Health and Nutrition Strategy 2006 to enhance the health and nutrition status of school-aged children. Despite these efforts, Nepal is in the top ten countries regarding stunting prevalence, a proxy for chronic malnutrition, and ranks among the top twenty in terms of stunted children, according to UNICEF 2009. (National Planning Commission, 2012).

**Fig. 4:**
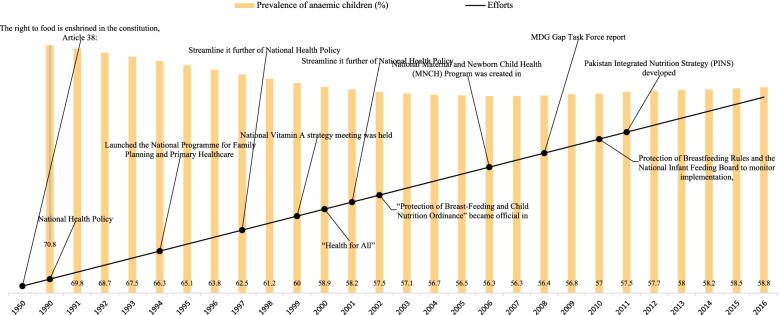
Prevalence of anemia among children aged 0–59 months and the chronology of various efforts to improve their nutritional status, Pakistan: 1950–2016

Figure 4 shows the prevalence of anaemia among children aged 0–59 months and the chronology of various efforts to improve Pakistan's nutritional status. Figure 4 manifests a 71% prevalence of anaemia among children in 1990. It also shows that 59%, 57%, and 59% prevalence of anaemia among children in 2000, 2010, and 2016. Only a 12% points reduction occurred in 26 years between 1990 and 2016 in Pakistan.

Nutrition has always been a low-priority area in Pakistan, with little political leadership involvement. Pakistan, on the other hand, reaffirmed its commitment to improving population health outcomes in 1978, when it signed the World Health Organization's Alma-Ata Declaration, which laid the groundwork for "Health for All" by 2000. (Turab, Tahir, & Zaidi, 2018). The right to food is established in Article 38 of the constitution. Additionally, the National Program for Family Planning and Primary Health Care, usually referred to as the Lady Health Worker Program was established under the umbrella of family planning in the 1990 National Health Policy. In 1990, anaemia was prevalent in children at a rate of 71%. After reviewing the 1990 National Health Policy, the government attempted to further simplify it in 1997 and 2001. The 1997 National Health Policy placed a premium on health promotion and education efforts. Pakistan could not reach the needed degree of development despite these attempts. Additionally, the 2008 MDG Gap Task Force report found that Pakistan continued to trail behind on key MDG indicators, with the United Nations (UN) identifying at least 24 indicators as being off track in 2008. These findings compelled politicians to establish the 2009 National Health Policy to address domestic issues and work toward achieving the Millennium Development Goals (GoP, 2009; Turab, Tahir, & Zaidi, 2018).

## Discussion

Malnutrition, impairs physical growth and development and, is a major contributor to increased morbidity and death in underdeveloped countries. Worldwide, two billion people are estimated to be malnourished. Childhood mortality due to various forms of malnutrition totals 2.6 billion, implying a massive worldwide illness burden. Malnutrition is very prevalent among Asian children, with Asia estimated to house 70% of the world's malnourished children.

The reasons for malnutrition are multifaceted since a vicious cycle of poverty contributes significantly to malnutrition's domination in some nations. Food insecurity disproportionately affects low socioeconomic status populations, and more disadvantaged households may be unable to get healthful meals owing to poverty. However, underlying drivers of malnutrition may vary among South Asia's underdeveloped nations. Because of the Sustainable Development Goals (SDG), these nations should establish a multifaceted strategy to prevent and manage malnutrition. The policy should address all facets, including dietary support, advocating for exclusive breastfeeding and appropriate complementary feeding, providing micronutrient fortification and supplementation, enhancing education, increasing nutritional awareness among adolescent girls and mothers, and ensuring complete immunisation coverage to prevent infectious disease, alleviating poverty, and ensuring food security.

Article 47 of the Indian constitution assigned the duty to the state to improve nutrition, the standard of living, and improve public health in 1950. The Indian government started several programs like a Mid-day meal program in primary schools by Tamil Nadu govt in 1962, the ICDS program in 1975, the Food for Work program in 1977, the Nutritious food scheme to reduce malnourishment- in Tamil Nadu in 1982, and the Mid-day meal by Kerala govt in 1984. After having constitutional rights and several national and state-level nutrition programs for 40 years, between 1950 and 1990, 77% of Indian children aged 0–59 months were anaemic. This plight situation might be the outcome of three primary reasons. Firstly, India’s engagement in several wars (Kashmir War 1947–48; India-China war of 1962, India-Pakistan war of 1965); Closed economy; lastly, India gained independence in 1947 only. After ten years, India got an eight percent reduction in anaemia from 1990 (77%) to 2000 (69%). During this period, the Indian govt made several programs like the Mid-day meal extended to 12 states in 1991, Feeding Bottles and Infant Foods Act in 1992, the Nutrition policy in 1993, and launched the Targeted Public Distribution System (TPDS) to resolve the problems of poor communities in 1997, etc. From 2001 (68%) to 2010 (60%), India again achieved an eight percent reduction in anaemia in children, which might be with the help of several policies like the extended Mid-Day Meal Scheme in 2002, National Health Policy in 2003, National Food for work (NFWP) in 2004, Adopted National Food Security Mission in 2007, National Rural Drinking Water Programme (NRDWP) in 2009, etc. India could not keep the reduction rate, after 2010, after making several policies like the National Rural livelihood Mission in 2011, National Policy for Children in 2013, National Food Security Act in 2013, Indira Gandhi Matritva Sahyog Yojana in 2014 etc. Therefore, India achieved only a two percent reduction from 2011 (59%) to 2016 (57%). The reason behind the low decline might be the problem in connection with the implementation of the programs.

Bangladesh became an independent country from Pakistan in 1971, unlike India. Bangladesh also gives food rights to its citizen by constitutional provision-article 18–1. Under this provision, Bangladesh had set up several institutions like the Institute of Public Health Nutrition to assist the government in formulating policy and strategies for nutrition-related activities and programs 1975, Bangladesh National Nutrition Council (BNNC) 1975. About 73% of Bangladesh children aged 0–59 months were anaemic in 1990 after 20 years of independence with constitutional food rights and several efforts. After ten years, Bangladesh reduced 11% anaemia from 1990 (73%) to 2000 (62%) through several efforts like the nutrition Policy and Programmed for Bangladesh, which was approved by the BNNC and published in Conference on Nutrition in 1992, participated in World Food Summit in 1996, National Food and Nutrition Policy in 1997, etc. From 2000 (62%) to 2010 (48%), Bangladesh again achieved a 14% reduction in anaemia in children, which might be with the help of effective policies like the National Food Policy in 2006 and previous policies. Bangladesh kept the reduction rate even after 2010 and again reduced eight percent of anaemia between 2010 (48%) and 2016 (40%).

Nepal attempted multiple measures to address nutritional problems in the country. It is a fundamental human right enshrined in the 'Convention on the Rights of the Child (CRC) 1989. After the National Nutrition Strategy in 1978. Further, Nepal National Plan of Action for Nutrition 1998, Nepal Health Sector Programme- Implementation Plan 2004–2009, National Nutrition Policy and Strategy 2004, National Plan for Action on Nutrition (NPAN) 2007, National Nutrition Policy and Strategy 2008, and current National Nutrition Program (Pahari, 2011) were implemented. The government has also implemented the School Health and Nutrition Strategy 2006 to improve school-aged children's health and nutrition status. Through these efforts, Nepal has reduced its anaemia by 31%, from 74% in 1990 to 43% in 2016.

Article 38 of the Pakistan constitution gives the right to food. After having constitutional rights for 40 years, between 1950 and 1990, 70% of Pakistan children aged 0–59 months were anaemic. This plight situation might be the outcome of two primary reasons. Firstly, Pakistan’s engagement in big wars with India (Kashmir War 1947–48; India-Pakistan war of 1965); secondly, Pakistan gained independence in 1947 only. After ten years, Pakistan got a 12% reduction in anaemia from 1990 (71%) to 2000 (59%). During this period, the Pakistan govt made several programs like National Health Policy in 1990, Launched the National Programme for Family Planning and Primary Healthcare in 1994, Streamline it further of National Health Policy in 1997, National Vitamin A strategy meeting held in 1999, and these policies might have played a vital role to get 12% anaemia reduction among children. From 2000 (59%) to 2010 (57%), Pakistan achieved only a two percentage reduction, clearly indicating child health priority changes prelude. Pakistan’s priority changes in child health have been crystal clear when its anaemia prevalence increased by two percentage from 2010 (57%) to 2016 (59%).

Selected countries have a constitutional base to provide nutritional support to their citizens; thus, India, Pakistan, Bangladesh, and Nepal have implemented nutritional programs. The prevalence of anaemia among children in 2016 shows that selected countries' efforts need to develop a comprehensive nutritional plan to deal with anaemia. India and Pakistan have a 70-year history, whereas Nepal and Bangladesh have a more than 50-year history of implementation, but anaemia prevalence is still high. These findings suggest that there is scope for strategy improvement.

Figures 1, 2, 3, and 4 reveals that all the countries had high anaemia among children in 1990. The prevalence of anaemia started declining in South Asian countries. Despite progress in the last decade for anaemia reduction in children, anaemia continues to be a significant public health concern in South Asia. Our findings have revealed that a holistic approach targeting the known underlying determinants of anaemia is needed to accelerate anaemia reduction.

The study finds that nutritional programs positively impact the prevalence of anaemia. The high prevalence was observed in 1990 and started to decline over time. A similar decline was observed in all the selected countries, except Pakistan. The majority of anaemia increased after 2010 in Pakistan, which shows a lack of interest in implementing the nutritional policy. On the other hand, the low prevalence was observed in Bangladesh and Nepal despite fewer policies and programs than in India and Pakistan, which manifests that the quality of policy is more important than the quantity of policy. The study suggests that India and Pakistan need to learn from the neighboring countries to eradicate anaemia among children.

The study compares nutrition policies and nutritional status of under-five children in four South Asian countries, namely Bangladesh, India, Nepal, and Pakistan. Although the reduction of anaemia is the outcome of many efforts, this study assumed that introducing exclusive nutrition policies and programs by four South Asian countries played an important role in anaemia reduction among under-five children since exclusive nutrition policies are meant to improve the nutritional situation.

## Conclusion

To conclude, the problem of under-nutrition in Pakistan and India stems from the lack of serious implementation of their National Nutrition Policies. Reducing anaemia among children requires solid political will to implement policies and programs. The silent under-nutrition crisis in Pakistan and India is quite alarming, particularly regarding children (the future of the society), and a government response is urgently required. The National Nutrition Policies must be revisited and modernized and should address the problem holistically with the latest demographic and epidemiological data. All selected countries urgently need the political will to manage anaemia through effective programs and satisfactory financial allocations that address the structural and systemic causes of anaemia. Policies should have monitorable targets, real-time monitoring and evaluation mechanisms, and accountability systems. In light of Sustainable Development Goals (SDG), a multi-faceted policy should be implemented to prevent and control malnutrition problems in these countries. Lastly, as we know, ministries work vertically (individually) in most countries, but we must understand that drafting and implementing the National Nutrition Program would require coordination between central ministries within a country to get the required level of success. Undoubtedly, a single ministry cannot grip this extremely multi-sectoral subject.

## Data Availability

Data has been taken from the Global Health Observatory Data Repository (GHODR), Demographic Health Survey (DHS) [https://dhsprogram.com/data/] and, and reviews some of the critical interventions in nutrition within the different countries and draws out several issues that bear on these policies' future evolution which are publicly available.
