# Correction: Nutritional policies and anaemia among under‑five children in selected south asian countries: 1950–2016

**DOI:** 10.1186/s12887-022-03652-0

**Published:** 2022-10-20

**Authors:** Hanumant Waghmare, Nasim Ahamed Mondal, Babul Hossain

**Affiliations:** 1 Department of Migration and Urban Studies, Post-Doctoral Fellow, International Institute for Population Sciences, Mumbai,, Maharashtra Pin: 400088 India; 2 Department of Social Sciences, Research Scholar, University of Warsaw, Warsaw, Poland; 3 Centre of Migration Research, Research Associate, University of Warsaw, Warsaw, Poland; 4 Doctoral Fellow, International Institute for Population Sciences, Mumbai,, Maharashtra Pin: 400088 India

**Correction:** ***BMC Pediatr*** **22, 540 (2022)**

10.1186/s12887-022-03597-4

In the originally published version of this article [1] two issues were found to be noted. First, the figures submitted earlier were not replaced by the shared modified figures file during the proofreading. Second, the alignment of the Table 1 (second column, column titled “Pooled”) in page 4 was not correct. The original article has been corrected.

## References

[1] Waghmare H, Mondal NA, Hossain B (2022). Nutritional policies and anaemia among under-five children in selected south Asian countries: 1950–2016. BMC Pediatr.

